# Effectiveness of dental whitening in patients with active fixed orthodontic treatment: a narrative review

**DOI:** 10.21142/2523-2754-1003-2022-119

**Published:** 2022-09-28

**Authors:** Adrián Mauricio Santillán-Guerra, Angie Melanie Sánchez-Rodríguez

**Affiliations:** 1 School of Dentistry, Científica del Sur University. Lima, Peru. amsg2502@gmail.com, angiesanchezrodriguez35@gmail.com Universidad Científica del Sur School of Dentistry Científica del Sur University Lima Peru amsg2502@gmail.com angiesanchezrodriguez35@gmail.com

**Keywords:** active fixed orthodontics, dental whitening, hydrogen peroxide, dental aesthetics, ortodoncia fija activa, blanqueamiento dental, peróxido de hidrógeno, estética dental

## Abstract

**Introduction:**

Currently dental aesthetics during fixed orthodontic treatment is an option required by patients, due to the frequent pigmentations that can occur in tooth enamel as a result of inadequate oral hygiene and plaque retention caused by fixed appliances.

**Objective:**

To determine the effectiveness of dental whitening and its side effects during active fixed orthodontic treatment, as an alternative aesthetic treatment in certain cases that merit it.

**Materials and methods:**

This narrative review evaluated 35 scientific articles from the Medline information sources (via PUBMED), EMBASE, SCOPUS, SCIELO and LILACS from the last 10 years, published in Spanish, Portuguese and English. We included 17 articles that met the selection criteria which were randomized clinical trials and observational studies, in vitro studies and in vivo studies that evaluated in-office dental clearance, performed on patients using fixed orthodontic treatment at that time. Systematic reviews, case reports and editorials were excluded.

**Results:**

It was observed that dental whitening in patients with active orthodontic treatment minimally affects the adhesion of the orthodontic apparatus to the tooth surface. In addition, some studies observed that the brackets interfere with the extension of the dental lightening, evidencing slightly darker areas.

**Conclusions:**

Dental whitening during orthodontic treatment can be given efficiently in clinical practice, depending on the aesthetic requirement of the patient. However, it is a recommended procedure in certain cases of great need for immediate aesthetics, since, the material with which the brackets are installed on the dental surface, will interfere with the extension of the lightening material, resulting in a darker shade in that sector and different from the surrounding tissue. Likewise, it will intervene in the adhesion to the dental surface, having many probabilities that the orthodontic apparatus can detach.

## RESUMEN

**Introducción:**

Actualmente, la estética dental durante el tratamiento de ortodoncia fija es una opción requerida por los pacientes, debido a las frecuentes pigmentaciones que se pueden presentar en el esmalte dental como consecuencia de una inadecuada higiene bucal y la retención de placa provocada por la aparatología fija.

**Objetivo:**

Determinar la efectividad del blanqueamiento dental y sus efectos secundarios durante el tratamiento de ortodoncia fija activa, como alternativa de tratamiento estético en determinados casos que lo ameriten.

**Materiales y métodos:**

Esta revisión narrativa evaluó 35 artículos científicos de las fuentes de información Medline (vía PubMed), Embase, Scopus, SciELO y LILACS de los últimos 10 años, publicados en español, portugués e inglés. Se incluyeron 17 artículos que cumplieron con los criterios de selección, los cuales fueron ensayos clínicos aleatorizados y estudios observacionales, estudios *in vitro* y estudios *in vivo* que evaluaron el aclaramiento dental en consultorio, realizados en pacientes que utilizaban tratamiento de ortodoncia fija en ese momento. Se excluyeron revisiones sistemáticas, informes de casos y editoriales.

**Resultados:**

Se observó que el blanqueamiento dental en pacientes con tratamiento de ortodoncia activa afecta mínimamente la adherencia del aparato de ortodoncia a la superficie del diente. Además, algunos estudios observaron que los *brackets* interfieren en la extensión del aclaramiento dental, al evidenciar áreas ligeramente más oscuras.

**Conclusiones:**

El blanqueamiento dental durante el tratamiento de ortodoncia se puede dar de manera eficiente en la práctica clínica, dependiendo del requerimiento estético del paciente. Sin embargo, es un procedimiento recomendado en ciertos casos de gran necesidad de estética inmediata, ya que el material con el que se instalan los *brackets* sobre la superficie dentaria interferirá en la extensión del material aclarante, lo que da como resultado una tonalidad más oscura en ese sector y diferente del tejido circundante. Asimismo, intervendrá en la adhesión a la superficie dentaria, por lo que existen muchas probabilidades de que el aparato de ortodoncia pueda desprenderse.

## INTRODUCTION

Dental whitening is a currently in-demand aesthetic treatment, which uses materials that react chemically with the tooth surface ^1^^,^^2^. The assessment of a harmonious smile with lightened teeth is highly considered by patients in a society very demanding of facial aesthetics ^3^^-^^5^. Even though the use of dental aligners has increased in orthodontic patients compared to conventional braces in search of less notoriety, there are societies where access to these, added to the biomechanical requirement of orthodontists of certain cases of malocclusion, make the use of fixed appliances continue to be considered an important alternative for this problem ^6^. For this reason, alternatives such as aesthetic braces or lingual orthodontics were developed. However, any aesthetic fixed appliance or not, will generate retention of bacterial plaque in different magnitude, depending on the oral hygiene of the patient, with the consequent side effects such as pigmentations in the enamel that are very rejected by patients.

The installation of fixed orthodontic appliances such as braces, implies an obstacle in the oral hygiene of patients, resulting in a decrease in salivary pH and an increase in bacterial adherence, due to the various electrostatic interactions due to the metal composition of these devices, thus stimulating an imbalance in the oral microflora ^6^. Also, to this factor are added aspects such as the type of adhesive; excess adhesive material; demineralization of enamel as a result of poor oral hygiene, tooth discoloration due to internal and external agents; unstable color of the type of resin used for the gluing of brackets or to the tooth surface, among other aspects that can change the hue of a tooth ^7^.

There are few studies that have evaluated the efficacy of whitening techniques in patients using fixed orthodontic appliances ^7^^,^^8^. Some of them were carried out in experimental studies on bovine teeth, however, their results cannot be extrapolated to humans ^9^. We also found a study ^9^ that evaluated the perception of orthodontists about the effect of dental whitening on teeth with brackets with encouraging results to be able to use this technique. However, there is no up-to-date review assessing the efficiency of clearance and its side effects in patients using fixed orthodontics ^9^. In some circumstances patients who wear braces require to have teeth lightened and the possibility of considering their dental aesthetics should clearly be studied. There are currently limitations on studies looking at the evidence on tooth whitening during orthodontic treatment. Therefore, the purpose of this study was to determine the efficacy of the dental whitening technique and its side effects during orthodontic treatment; as a sporadic treatment to improve the aesthetics of patients with fixed orthodontics.

## MATERIALS AND METHODS

**Figure 1:**
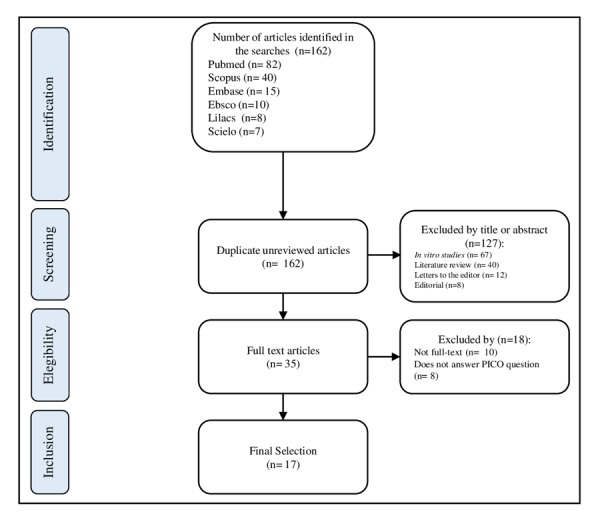
PRISMA Flow chart of the literatura review

A narrative review was carried out that evaluated 36 scientific articles from the information sources MEDLINE (via PUBMED), EMBASE, SCOPUS, SCIELO and LILACS from the last 10 years, published in Spanish, Portuguese and English. After a selection of studies by title and abstracts, only 17 articles that met the selection criteria including randomized clinical trials and observational studies were included. They evaluated the dental whitening in the office, carried out on patients who were using fixed orthodontic treatment at that time. Systematic reviews, case reports and editorials were excluded. Figure 1. 

## EFFICACY OF DENTAL WHITENING IN PATIENTS WITH BRACKETS.

The outer layer of the dental organ is made up of a mineralized tissue called tooth enamel, which has most of its mineral composition of hydroxyapatite that form the prisms of enamel and to a lesser extent has organic elements ^7^^,^^10^. Any change in this substrate will have a direct relationship with the adhesion of the brackets, since that is where they adhere and start the orthodontic treatment ^11^.

On the other hand, it is common for fixed orthodontic treatment to present as a side effect a dental pigmentation, due to poor oral hygiene habits of the patient ^12^, or by the overflowing adhesion materials themselves that generate bacterial plaque retention, or by the existing corrosion of the metal brackets due to a decreased oral pH and the diet rich in acids and dyes ^13^^,^^14^.

Dental whitening is an alternative of aesthetic treatment rare in this active phase of orthodontic treatment, and seeks to solve the darkening or pigmentations of the teeth with its consequent improvement in the dental aesthetics of the patient ^3^^,^^9^^,^^13^^,^^15^^,^^16^. Dental whitening is feasible at this stage because its active ingredient contains molecules of low molecular weight, allowing the material to spread over the entire surface of this hard tissue avoiding the whitening only of the contour of the enamel where the bracket was located and achieving a continuous tonality that can be observed after the removal of the brackets ^7^^,^^17^^-^^19^. The whitening mechanism has evidenced the division of hydrogen peroxide into oxygen and perhydroxyl radicals that contribute to the elimination of the chains belonging to the substances that cause the change of color of the tooth, also called chromophores, resulting in the whitish appearance of the dental tissue ^13^^,^^17^^,^^20^.

Usually, the dental whitening procedure is performed after orthodontic treatment.^7^ However, in some circumstances the motivation of the patient in wanting to present a smile aligned at the same time with teeth clarified, led to the report of in vivo and in vitro studies, which show that dental whitening during an active fixed orthodontic treatment is an apparently non-harmful and effective aesthetic alternative ^7^^,^^8^^,^^21^.

They were able to find in vitro research using bovine teeth, which are a substrate very similar to human teeth. However, they lack factors typical of the human oral cavity, such as saliva, the patient's dental hygiene, the reaction of adhesion products in the dental substrate, dental pH ^3^^,^^21^, which hinder their ability to extrapolate data and add it to dental clinical practice, although a reference can guide. In this sense, an in vitro study concluded that dental whitening with carbamide peroxide in bovine teeth is effective during and after orthodontic treatment, although changes were evidenced over time ^22^. Another study, evaluated the efficacy of dental whitening with carbamide peroxide during orthodontic treatment by spectrophotometry in bovine teeth, concluding greater effectiveness of home whitening over whitening in office ^11^, they also concluded that the homemade whitening technique presents better results, because of the long exposure that the material presents in the teeth and that also influences the percentage of concentration of the compound active ^8^^.^^23^. In general, the interference of the brackets had no relevance in the dental whitening, after two weeks in tests with bovine teeth, evidencing that the adhesive material does not hinder the uniformity in the whitening ^3^.

This type of procedure has also been studied in humans. One of them sought to generate a dental whitening protocol during orthodontic treatment, concluding that this treatment is successful, despite the presence of brackets on the tooth surface ^7^. Another compared new lightening products, such as 8% and 10% hydrogen peroxide in order to measure their effectiveness and sensitivity generated by these materials, evidencing a high efficiency and low sensitivity ^16^.

Currently, there are few studies that can corroborate the complete efficacy of dental whitening during orthodontic treatment. Despite this, the studies evaluated concluded that this modality can be included as a clinical alternative in the patient's dental aesthetics, complementing greater dental and facial aesthetics ^7^^,^^16^.

## ADVANTAGES AND DISADVANTAGES OF DENTAL WHITENING IN PATIENTS WITH BRACKETS.

In orthodontics, dental whitening is usually performed after the removal of the brackets, but in some cases, when the patient requires it and there is still time to finish the treatment, there is the possibility of performing it with active orthodontic appliances ^24^. Therefore, it is important to know the mechanism of action of bleaching products on the tooth surface and the techniques to achieve this, such as the in-office technique and the home technique. Home bleaching agents are applied in low concentrations and have a longer duration than in-office clearance; in which they use high concentrations in short periods, however, they need buckets that will restrict tooth movement, so it is not an alternative for these cases ^25^. In this sense, the ideal alternative for these patients who request this condition with haste, the office technique is the one that would manage to clarify the teeth of these patients.

This whitening during orthodontic treatment turns out to be a motivational determinant, which he attributes to the patient's confidence in order to achieve a smile with clear and aligned teeth. Orthodontic treatment is known to be extensive and the opportunity to lighten teeth in conjunction with orthodontic treatment increases patients' expectations, as this will save time, as long as the patient cooperates with very efficient oral hygiene ^7^.

Dental whitening depends on a correct penetration of the clarifying agent and its by-products throughout the tissue. When the goal is an ideal whitening, it should be borne in mind that this is not during orthodontic treatment, it should only be left for emergency circumstances, since there is a risk that it can fail quickly and darken the tone of the tooth by the use of the brackets, since these retain plaque that can pigment the teeth. In addition, in the phase of removal of brackets some variation of the texture of the enamel can be generated if the technique of removing brackets was not ideal. In addition, the enamel of whitened teeth may be weaker after whitening ^8^^,^^18^^,^^26^.

Another disadvantage of lightening during orthodontic treatment is that the pores of the enamel surfaces are open, and for this reason the union of the resin with the tooth loses rigidity; that is why brackets are unlikely to detach from the tooth surface ^18^.

According to some authors ^3^^,^^8^^,^^19^^,^^27^, it is very likely that the marks of the binding agent in this case the resins, hinder the expansion and action of the bleaching agent, this could cause a probability of different color in the tooth after removal of brackets.

An in vitro study showed that both whitening procedures, both the office and the home, reduce the binding force of orthodontic appliances to tooth enamel. This study also concludes that the clearance in the area under the brackets also achieved clearance, but with a lower intensity than the remaining enamel. That is why patients who require this type of treatment should be informed as they are likely to need a second clearance treatment after orthodontics ^8^.

In another study they compared the types of aesthetic brackets, among them (monocrystalline and polycrystalline), where they concluded that monocrystalline brackets showed higher shear resistance (RC) values compared to polycrystalline, and it is explained that it is due to their composition and manufacture, they also relate it to the fact that it has less incorporation of impurities, which makes the support more translucent thus allowing the support to retain less light, increasing retention in the tooth ^28^.

One study evaluated a protocol for joining metal brackets after whitening with hydrogen peroxide, and concluded that the antioxidant treatment applied immediately after whitening was effective in reversing the reduction in shear resistance of the brackets after teeth whitening, in this case they used 10% sodium ascorbate, which reversed the decrease in shear resistance ^21^^,^^27^. Likewise, another study demonstrated the reduction of the shear resistance of composite resin adhered to the enamel followed by the clearance with hydrogen peroxide to 35%, in addition to stating that 10% of sodium ascorbate increased the bonding force after clearance with respect to ceramic brackets and almost the same result for metal brackets ^29^. In relation to the method of clearance and resistance to shear, a study concludes that the whitening with the home method affects the SBS significantly more than the method in office or office, because the bleaching gel in the home method has a longer exposure time in the teeth which causes internal alterations ^30^.

In general, some effects of whitening are the adhesive reduction of the brackets to the tooth surface, white spots, irregularity of the color of the enamel when removing the orthodontic appliance, retention of bacterial plaque and a probable detachment of some superficial part as a result of dental whitening during orthodontic treatment, because the clarifying agents that are used generate the demineralization of tooth enamel ^18^.

That is why associating dental whitening during the use of orthodontic appliances is still a controversy, but it can indeed be done as a very urgent alternative at the request of the patient, in very special cases of high aesthetic requirement. In addition, it must be performed by a trained professional and in specific cases.

Finally, this topic is very little explored, therefore, more studies should be done to ensure a correct and safe indication for the patient.

## DISCUSSION

Dental whitening in patients with fixed orthodontics is a recently used aesthetic alternative, as a result of the patient's requirement to look good ^3^^,^^9^^,^^13^^,^^15^^,^^16^. Information was obtained to support this procedure as an effective and recommended alternative in some cases ^7^. Likewise, in vitro studies concluded that, during an orthodontic treatment, this aesthetic procedure performed with carbamide peroxide in bovine teeth was effective. However, the whitening results obtained with the group of teeth with brackets were not very significant in the first weeks or months of control ^22^, since, factors such as intrinsic and extrinsic stains on the tooth surface and the constant formation of repairing dentin by the pulp tissue, were causal for the low effect of aesthetic treatment ^31^.

In this stage of orthodontic treatment, it is feasible to think about performing a dental whitening, since the whitening material has low molecular weight, which will contribute to its extension, giving as a clarifying effect on the entire dental surface ^7^^,^^17^^-^^19^. Despite this, an in vitro study where they evaluated by spectrophotometry bovine teeth with orthodontic treatment, contradicts this theory, since, their results observed that the dental surface had clarified except for the sector where the bracket was placed ^11^.

On the other hand, an in vitro study carried out on bovine teeth made a comparison between the whitening of the teeth during orthodontic treatment with those that do not present this type of treatment in two weeks, concluding that there was not much difference in the results obtained, despite the thickness of the material placed of the cementing resin of the bracket ^3^. Likewise, an in vivo study managed to evaluate the effectiveness of 8% hydrogen peroxide in patents with fixed orthodontic appliances, observing that dental whitening in carrier and non-carrier patients was very effective and similar ^32^. However, the time in which they perform the controls is very short so that these studies can be reaffirmed and extrapolated in clinical practice.

Shear resistance is a relevant topic within the clearance during orthodontic treatment, since it is related to the binding agent and the tooth. An in vitro experimental study demonstrated a decrease in the shear binding strength of brackets after clearance ^13^. In relation to CR, there are several studies that have shown the decrease in CR related to clearance during orthodontic treatment ^30^^,^^33^. In addition, the result of an in vitro study showed that both lightening techniques (home and office) decrease the binding force of orthodontic braces to enamel ^8^.

With respect to the different materials used to decrease CR during and after clearance, several studies show that antioxidant treatment and other materials used after clearance are effective in increasing the shear resistance of brackets ^21^^,^^27^^,^^34^^,^^35^. The presence of a bracket attached to the tooth acts as a barrier to the penetration of peroxide, which affects the homogeneity of color in the samples of this research ^24^. The investigation of another in vitro study presented some change in the tonality in the area under the bracket, since it did not have direct contact with the lightening agent, which would ratify the aforementioned study ^3^.

In addition, a study conducted on bovine teeth found that the presence of the binding agent acted negatively on the efficiency of both office and home clearance and showed that home dental whitening is more effective than that performed in the office ^11^. On the contrary, one article relates that hydrogen peroxide penetrates strongly on tooth enamel, and is probably the most intense element in penetration into dental structures. If used directly in the enamel to which a bracket adheres, hydrogen peroxide can access the subsoil of the enamel and thus comes to clarify the enamel area that is covered by the bracket ^18^. Finally, a study shows that both areas will have a lightening effect, but they report that the enamel that is lower than the bracket has a lower lightening effect than the rest of the enamel discovered ^8^. Tooth enamel after tooth whitening can present a more fragile structure ^8^, that is why it is associated with a possible detachment of the brackets.

Dental whitening during active orthodontic treatment is a procedure that has advantages and disadvantages; so, it is necessary to perform it in particular cases or in patients with high aesthetic expectations. The clinical evidence is positive and encourages an alternative as an interesting treatment for patients and health professionals. For these reasons, further research is suggested to investigate whether the clearance procedure during orthodontic treatment presents damage and what are the contraindications to not performing it.

## CONCLUSIONS

Dental whitening during orthodontic treatment can be given efficiently in clinical practice, depending on the aesthetic requirement of the patient. However, it is a recommended procedure in certain cases of great need for immediate aesthetics, since, the material with which the brackets are installed on the dental surface, will interfere with the extension of the lightening material, resulting in a darker shade in that sector and different from the surrounding tissue. Likewise, it will intervene in the adhesion to the dental surface, having probabilities that the orthodontic apparatus can detach.

## Financing Sources:

Self-financed
